# Key Performance Indicators of Chest Pain Management in Emergency Department; a Letter to the Editor

**Published:** 2018-02-18

**Authors:** Mehrdad Taghizadeh, Roghayeh Taghipour, Kamran Heydari

**Affiliations:** 1 Emergency Department, Loghman Hakim Hospital, Shahid Beheshti University of Medical Sciences, Tehran, Iran.

**Keywords:** Electrocardiography, thrombolytic therapy, myocardial infarction, early diagnosis, delayed diagnosis; time-to-treatment

**Dear editor:**

Chest pain is a common complaint among those presenting to emergency department (ED) and is associated with a high rate of mortality. Based on National Center for Health Statistics, chest pain leads to about 6 million visits to EDs in United State. In Iran, ischemic cardiac diseases are the second cause of death in people aged 15 to 49 years (1).

While less than 10% of the patients presenting to ED with chest pain are affected with myocardial infarction (MI), 33% of MI cases are silent (2). Timely diagnosis and re-vascularization of ischemic part, using thrombolytic agents or percutaneous coronary intervention (PCI), can save cardiac function and the patient’s life (3).

Therefore, in this regard the emphasis is on rapid referral of the patients to treatment centers, initiation of diagnostic measures such as electrocardiography (ECG) and in case of meeting the required criteria, transfer to cat lab for PCI. American Heart Association (AHA) has introduced the proper door to ECG time as 10 minutes if it has not been done in the ambulance (4). Based on this recommendation, AHA has defined the aims of coronary reperfusion as prescription of thrombolytic in the initial 30 minutes after admission of the patient to ED or PCI in the initial 90 minutes after admission to ED.

Efficacy of reperfusion therapy decreases with an increase in the time interval between manifestation of symptoms and initiation of treatment. Potential delay in initiation of reperfusion therapy can be due to increase in Door to Data, Data to Decision, and Decision to Drug times. Therefore, improvement of these times as a series of Key Performance Indicators has always been of interest to health care managers.

By performing a clinical audit on 781 patients with the mean age of 50.91 ± 16.76 (20 -93) years who were included via non-probability census sampling and had presented to ED of Loghman Hakim Hospital, Tehran, Iran, during 1.5 years with the chief complaint of chest pain (53.3% male), the writers of the present letter estimated mean and standard deviation of door to first ECG performance time as 22.47 ± 16.59 (0 – 94) minutes. Time interval between admission of the patient to receiving thrombolytic in this study was 46.60 ± 17.98 (22-82).

Mean and standard deviation of the time interval between ECG to making a decision for starting thrombolytic, for 23 patients with ST segment elevation myocardial infarction (STEMI), was 26.90 ± 18.15 (0 – 65) minutes. In addition decision time to injection of thrombolytic in this study was almost 0 since decision making was done in ED while the drug was present at the patient’s bedside.

There was no significant difference between various working shifts including morning (22.62 ± 16.01 minutes), evening (22.15 ± 16.61 minutes), and night (22.24 ± 17.33 minutes) in this regard (p = 0.827). The final diagnosis was unstable angina for 151 (19.3%) cases, MI in 32 (4.1%) cases (23 STEMI cases), and pulmonary embolism in 2 (0.3%) cases.

As can be seen, door to first ECG time in the studied ED is approximately twice the standard time proposed by AHA. In addition, door to drug time interval is also about 1.5 times more than the mentioned standard.

Managers of the mentioned ED attempted to relieve the problem by increasing the number of ECG devices and putting someone in charge of performing ECG for each shift. In addition, by training triage nurses it was decided that all the patients presenting to ED with complaint of non-traumatic chest pain, either typical or atypical, should undergo screening immediately using ECG in the fast track and their ECG should be interpreted by the senior resident in each shift. Additionally, the method of recording the mentioned times was changed from manual, which was estimated and recorded with 3 to 5 minutes delay from the time of initiation of the measures by a nurse, to automatic with calibrated ECG devices.

Overall, it seems that improvement of the mentioned times needs constant care of the ED managers and continuous training of triage and EMS personnel for acceleration of providing diagnostic and treatment services to these patients. It should be noted that fortunately by setting up “724 system” by the ministry of health and performance of ECG in the ambulance and transmitting the data from the ambulance to the dispatch center, this problem will be solved soon. 
